# Association Between Impacted Mandibular Third Molars and Temporomandibular Dysfunction: An Analysis Based on the Modified Helkimo Index

**DOI:** 10.3390/medicina61050850

**Published:** 2025-05-05

**Authors:** Dorin Ioan Cocoș, Alexandru Vlasa, Sorana Maria Bucur, Mariana Păcurar, Kamel Earar

**Affiliations:** 1 Faculty of Dental Medicine, “Dunărea de Jos” University, 800008 Galati, Romania; cdorin1123@gmail.com (D.I.C.); kamel.earar@ugal.ro (K.E.); 2 Department of Periodontology and Oral-Dental Diagnosis, George Emil Palade University of Medicine, Pharmacy, Science, and Technology of Târgu Mureș, 38 Ghe. Marinescu Street, 540139 Târgu Mureș, Romania; alexandru.vlasa@umfst.ro; 3 Department of Dentistry, Faculty of Medicine, “Dimitrie Cantemir” University of Târgu Mureș, 540545 Târgu Mureș, Romania; 4 Department of Orthodontics, George Emil Palade University of Medicine, Pharmacy, Science, and Technology of Târgu Mureș, 38 Ghe. Marinescu Street, 540139 Târgu Mureș, Romania

**Keywords:** temporomandibular dysfunction, Modified Helkimo Index, impacted third molars, occlusal imbalance

## Abstract

*Background and Objectives*: To evaluate the impact of impacted mandibular third molars on temporomandibular joint dysfunction using the Modified Helkimo Index, analyzing symptom severity across age groups. *Materials and Methods*: A cohort of 140 patients (70 with impacted molars, 70 without) was assessed using the Modified Helkimo Index. Patients were categorized by age (<25, 26–30, 31–35, >36 years), and statistical comparisons between Icd^i^ (with impacted molars) and Icd^a^ (without impacted molars) were performed. Key parameters included mandibular movement limitation, joint noises, and pain scores. Data were analyzed using descriptive statistics and statistical tests, with significance set at *p* < 0.05. *Results***:** TMJ dysfunction was significantly more prevalent in patients under 25 years (Icd^i^ = 13.5, Icd^a^ = 11.0; *p* = 0.045), with a progressive decrease in severity in older groups (>36 years: Icd^i^ = 3.5, Icd^a^ = 4.5; *p* = 0.072). Women exhibited a higher prevalence across all age categories (female-to-male ratio: <25 years = 2.7, >36 years = 3.0). The most frequent symptoms were mandibular movement restriction (42.5%), joint noises (38.2%), and pain (35.7%). *Conclusions*: Impacted third molars may significantly exacerbate TMJ dysfunctions, particularly in younger individuals and females, with a strong association between impacted molars and increased Modified Helkimo Index scores. Early extraction might mitigate symptoms, emphasizing the need for proactive clinical management.

## 1. Introduction

The temporomandibular joint (TMJ) is a pivotal articulation connecting the mandible to the temporal skull bone, anterior to each ear. This joint facilitates essential movements such as opening and closing the mouth, chewing, and speaking due to its unique structure that combines hinge and sliding motions [1]. Temporomandibular disorders (TMDs) encompass a range of conditions affecting the TMJ, masticatory muscles, and associated structures. The etiology of TMDs is multifactorial, involving malocclusions, such as open bites or significant overjets, that can predispose individuals to TMDs [1,2].

Trauma, such as direct injury to the jaw, and microtrauma, repetitive behaviors like teeth grinding and clenching, can initiate TMDs. Stress, anxiety, and depression have been linked to increased muscle tension and parafunctional activities, contributing to TMDs. Factors such as poor health, nutrition, joint laxity, and exogenous estrogen can influence the development of TMDs [1,2]. Epidemiological studies indicate that TMDs affect a significant portion of the population. A comprehensive meta-analysis reported an overall prevalence of approximately 31% in adults and older persons, with disk displacement disorders being the most common subtype [1].

Most TMD patients who sought treatment were between 16 and 35 years old. There were approximately twice as many female patients as male patients. On average, females delayed seeking care longer than males and were slightly older when they did. Jaw movement limitations were more commonly reported among female patients [2].

These findings underscore the importance of recognizing the multifaceted nature of TMDs and the need for tailored approaches in diagnosis and management.

Impacted mandibular third molars are not a well-documented risk factor for TMD. Although several studies have examined TMD in the context of impacted molars, few have employed rigorous clinical and statistical methodologies to establish a direct relationship using a well-defined patient sample.

The relationship between TMJ pain and wisdom teeth extractions, particularly impacted third molars, was explored [3]. The complications that can arise from the extraction of these molars, such as persistent TMJ discomfort, and their association were investigated. Although most post-surgical issues are resolved within a few weeks, some patients may experience longer-term pain due to trauma during the extraction process [2,3]. The research highlights the need for careful evaluation and management of patients undergoing wisdom tooth removal, as the proximity of impacted third molars to the TMJ can exacerbate or trigger TMD symptoms [3]. It emphasizes the importance of early identification and preventive measures to reduce the risk of TMJ damage and improve patient outcomes.

The relationship between craniofacial morphology, orthodontic needs, and TMD was studied, suggesting that stronger bite forces are still present despite the orthodontic or TMD concerns [4].

The potential association between impacted mandibular third molars and TMD remains critical in dental research. The relationship between third molar impaction and TMD was analyzed in 80 patients (aged 20–60 years) with impacted mandibular third molars. Clinical and radiological evaluations revealed that TMDs were most prevalent in cases of horizontal impaction (63.8%), followed by mesioangular (46.3%), distoangular (42.5%), and vertical impaction (30.0%) [5]. These findings suggest that the type of impact may influence the severity of TMD, emphasizing the need for early diagnosis and targeted interventions. Understanding this correlation is essential for optimizing clinical management, preventing long-term joint damage, and guiding prophylactic treatment strategies for at-risk patients. Further large-scale, multi-center studies are needed to validate these associations and refine treatment protocols.

Beyond localized pain and pericoronitis, impacted third molars may alter dental occlusion and might increase muscle tension by affecting the craniofacial system. Chronic inflammation linked to impacted molars could predispose patients to TMD by inducing structural and functional disturbances in the stomatognathic system, as suggested by previous studies [3,5,6,7].

The TMJ, a complex synovial joint, enables essential movements for mastication, speech, and swallowing. It consists of the mandibular condyle, temporal bone fossa, and an interposed articular disc, allowing rotational and translational motions to adapt to occlusal forces [8,9]. Dysfunction of this joint presents as pain, joint noises (clicking, crepitus), limited mandibular movement, and muscle spasms, often interfering with daily activities. TMD predominantly affects women, with a peak incidence between 20 and 40 years of age [10,11].

Since TMJ function is closely integrated with the teeth, masticatory muscles, and central nervous system, disturbances in any of these components can contribute to TMDs [9]. Impacted third molars, due to their anatomical positioning, have a strong influence on occlusion and mandibular dynamics, potentially leading to dysfunction [12]. The resulting chronic pain and mobility restrictions significantly impact the quality of life [9,10].

The biomechanical influence of impacted molars has been proposed to extend beyond dental alignment. The pressure exerted on adjacent teeth may induce occlusal imbalances, causing excessive strain on the masticatory muscles and inducing abnormal mandibular movements [13,14]. This, in turn, could result in muscle fatigue, chronic pain, and restricted mandibular function, particularly affecting the masseter and temporalis muscles [14,15].

Joint noises such as clicks and crepitations, commonly reported in TMDs, might be aggravated by deviations in mandibular movement related to the impacted molars [16,17]. Moreover, pain from the affected molars may radiate to the TMJ, mimicking the dysfunction’s symptoms and complicating accurate diagnosis if the underlying cause is not correctly identified, as TMJ pain can come from multiple sources [5,11,17]. Additionally, chronic pain associated with TMDs may lead to psychological comorbidities such as stress, anxiety, and depression, further exacerbating symptoms [18,19,20,21].

The Modified Helkimo Index (Table 1) is a standardized clinical tool for assessing TMD severity [22]. The Helkimo Index was chosen over the DC/TMD due to its simplicity and focus on evaluating dysfunction severity, including joint mobility, pain, and muscle sensitivity. While the DC/TMD is more suited for detailed diagnostic classification of TMD subtypes [23], the Helkimo Index provides a structured and consistent method to analyze the severity of dysfunction associated with impacted third molars, aligning with the objectives of this study. Our study aims to bridge this gap by evaluating the influence of impacted mandibular third molars on TMD severity using the Modified Helkimo Index.

Due to the inconclusive and varied evidence in the current literature regarding the correlation between impacted third molars and TMD [14,15,16,17], this research is necessary to provide insights and contribute new data on the potential impact of impacted molars on TMJ health.

By stratifying patients based on age and gender, we provide a detailed analysis of the association between impacted molars and TMD, contributing to improved diagnostic and therapeutic strategies.

The null hypothesis (H_0_) stated that there was no significant association between impacted mandibular third molars and TMDs. We assume by this hypothesis that the presence of partially impacted mandibular third molars had no significant effect on the prevalence or severity of TMDs, as measured by the Modified Helkimo Index, and that occlusal imbalances, muscle strain, and inflammatory responses do not significantly contribute to TMJ pathology in affected individuals.

## 2. Materials and Methods

Our descriptive study was conducted under the Declaration of Helsinki and approved by the Institutional Review Board (or Ethics Committee) of ”Dunărea de Jos” University (no. 16/CEU/2024, 9 December 2024).

The study included a cohort of 140 patients, of whom 70 presented a partial inclusion of the wisdom tooth in mesioangular, distoangular, or vertical positions, and the remaining 70 were without any inclusion of the wisdom tooth. This selection method correlates the presence or absence of the partially erupted wisdom tooth with the presence of TMJ pathology using the modified Helkimo index.

The cohort was divided into subgroups based on age: under 25, 26–30, 31–35, and over 36. This group of patients was analyzed to determine if the inclusion of the wisdom tooth has implications for TMJ pathology.

The Modified Helkimo index was used to detect TMD. The software used for data processing is Python, version 3.9.

The Modified Helkimo Index scale was used to evaluate the severity of TMDs [22]. The original Helkimo Index is a clinical tool used to assess the severity of TMDs. Developed by Martti Helkimo in 1974, this index has undergone various modifications by researchers over the years to improve its applicability and accuracy. One significant adaptation was Maglione, who enhanced the index’s diagnostic utility [22].

Table 2 illustrates the classification of the severity of TMDs according to the Modified Helkimo Index.

This classification provides a structured way to assess the severity of TMDs based on the patient’s score [22].

For the evaluation of patients using the Helkimo index, the analysis was based on the examination of two sub-groups, correlated with third molar inclusion:

I. Partial inclusion of the wisdom tooth in mesioangular, distoangular, or vertical positions—Icd^i^;
II. The absence of partial inclusion of the wisdom tooth—Icd^a^.

We used the Helkimo index to assess key parameters such as mandibular movement range, muscle and joint sensitivity, joint noises, and pain associated with jaw movements. The 140 patients were evaluated using the two sub-indices: Icd^i^ (the clinical dysfunction index of the TMJ correlated with impacted molars) and Icd^a^ (the clinical dysfunction index of the TMJ without impacted molars).

Inclusion Criteria

- Patients aged between 18 and 65 years old.
- The presence or the absence of partial inclusion of the lower wisdom teeth confirmed by clinical and imaging investigations.
- Willingness to participate in the study, complete clinical evaluations, and Helkimo index scoring.
- Signing the agreement and informed consent form.

Exclusion Criteria

- Patients with pre-existing TMJ pathologies (malignant/benign lesions).
- History of severe trauma to the condylar joint or the mandibular body.
- Wearers of orthodontic appliances or undergoing orthodontic treatment.
- Patients with severe systemic conditions (e.g., rheumatoid arthritis) that may affect the TMJ function.
- Recent use of medications that may mask the symptoms of TMD (e.g., painkillers, muscle relaxants).
- Refusal to participate or sign informed consent.

All statistical analyses were performed using Python (version 3.9). Data were tested for normality using the Shapiro–Wilk test. Comparative statistical analyses between the Icdi (patients with impacted third molars) and Icda (patients without impacted third molars) groups were conducted using independent sample *t*-tests for normally distributed data and Mann–Whitney U tests for non-normally distributed variables. Categorical variables were analyzed using the chi-square test.

To assess the severity of TMDs, mean values, standard deviations (SDs), and 95% confidence intervals (CIs) were calculated for Helkimo Index scores across age groups. Statistical significance was set at *p* < 0.05. Additionally, effect sizes (Cohen’s *d*) were computed for significant comparisons to determine the magnitude of differences between groups, with values of 0.2, 0.5, and 0.8 representing small, medium, and large effects, respectively (Cohen, 1988) [24].

## 3. Results

**Figure 1:**
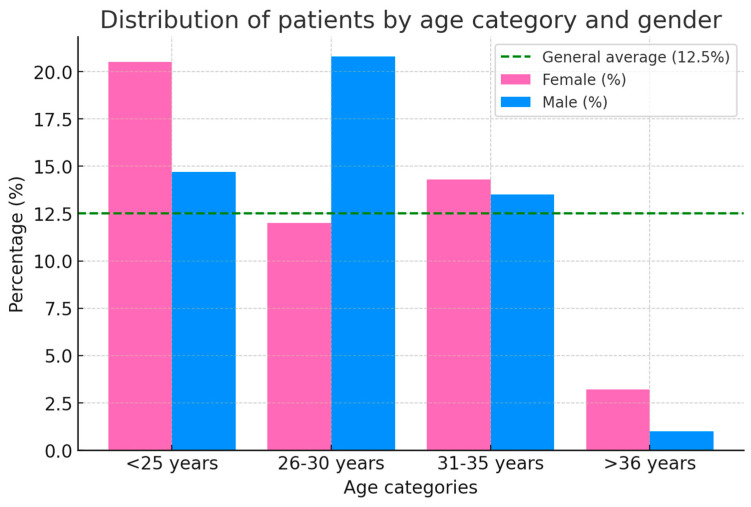
The distribution of patients is segmented by age and gender.

This study included 140 patients (93 females and 47 males) distributed across four age categories: <25 years, 26–30 years, 31–35 years, and >36 years (Figure 1). The following tables and figures present detailed demographic and clinical data, including statistical significance and effect sizes. Figure 1 illustrates the distribution of patients segmented by age categories and gender. It shows a higher prevalence of TMJ pathology in females across all age groups, with a peak at under 25 (38 women and 14 men). The number of cases decreases progressively with age, with the lowest recorded in individuals over 36.

Table 3 presents gender distribution across age categories. The patients were segmented into age categories and analyzed separately in the female and male groups, indicating both the absolute number of cases (“n”) and their proportion (%) within each respective group. Additionally, the female/male ratio is calculated for each age category. The results highlighted a higher prevalence of inclusion in females, regardless of age. While a higher proportion of females was observed in all groups, no statistically significant gender difference was found in any category (*p* > 0.05).

**Figure 2:**
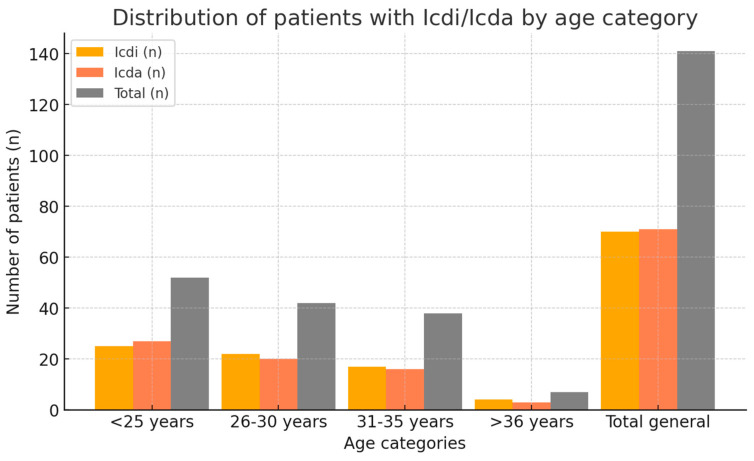
Distribution of patients with Icd^i^/Icd^a^ across age categories.

Figure 2 illustrates the distribution of patients with impacted (Icd^i)^ and non-impacted (Icd^a^) third molars across age groups. The categories analyzed are: under 25 years, 26–30 years, 31–35 years, and over 36 years. The graph highlights the differences between the groups, providing a clear perspective on the distribution. It is for visualizing variations by age and understanding the general proportions within each analyzed subgroup. Younger age groups (<25 and 26–30) had higher frequencies of impacted molars than older age groups.

Table 4 presents the distribution of patients with Icd^i^/Icd^a^ across age categories. The columns include the number and percentage of patients included in the study and the total number of patients. These data provide a demographic distribution and help comparative analysis between age categories.

**Figure 3:**
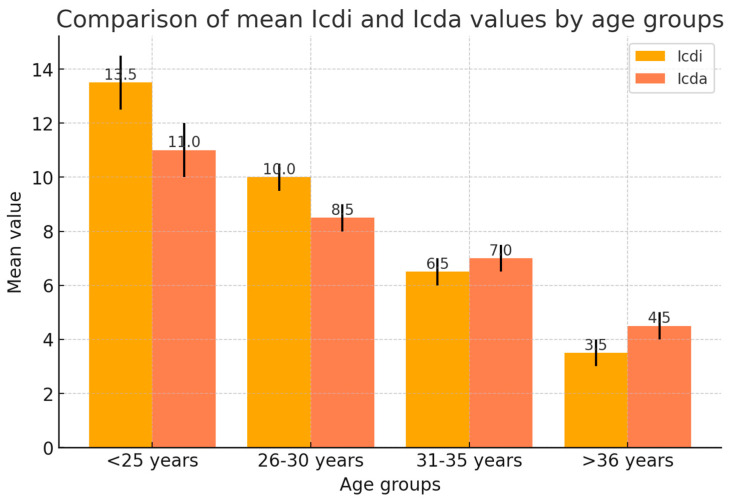
Distribution of patients with Icd^i^/Icd^a^ across age means.

Figure 3 compares mean Helkimo Index values for patients with Icd^i^ (in the presence of impacted molars) and Icd^a^ (without impacted molars) according to age categories. The bars represent the mean values, while the error lines indicate 95% confidence intervals for each group. A noticeable difference in mean scores was observed in younger groups. Icd^i^ values are consistently higher across all age groups, with significant differences observed in younger patients (<25 years and 26–30 years). The decrease in values with age suggests a reduced impact of impacted molars on symptoms in older patients.

Table 5 compares Helkimo Index scores between groups with and without impacted third molars. It includes the mean, minimum, and maximum values for Icd^i^ (with impacted wisdom teeth) and Icd^a^ (without them) and the 95% confidence intervals. Statistically significant differences were observed in patients under 30, with medium-effect sizes indicating clinically relevant differences. The *p*-values indicate the statistical significance of the difference between Icd^i^ and Icd^a^. The data highlight variations in the prevalence and severity of TMD symptoms based on age and the presence of impacted molars, providing essential insights for clinical assessment and personalized patient management.

## 4. Discussion

This study presents a novel and integrated approach to a frequently encountered but insufficiently explored issue in dental practice: the relationship between impacted lower third molars and TMDs. By utilizing the Modified Helkimo Index, the study provides an objective and measurable framework for assessing the influence of impacted molars on TMJ pathology, enabling precise classification of patients based on symptom severity. Additionally, segmenting the subjects by age and gender allows for a more detailed analysis of specific prevalence patterns [25].

In line with the existing literature, our study confirms the sex- and age-related differences in the prevalence and severity of TMDs [4]. The results confirm a higher occurrence of TMD among female patients across all age categories, with the highest incidence in the <25-year group. The female-to-male ratio remains elevated in all age groups, peaking at 3.0 in individuals over 36 years [26]. These findings align with the existing literature, which suggests increased susceptibility in women due to hormonal, anatomical, and behavioral factors such as estrogen fluctuations, increased joint laxity, and stress-related parafunctional habits like bruxism [26]. According to previous studies, it has been shown that sleep bruxism plays a key role in TMDs, particularly in women, where its prevalence increases with age. This trend is not observed to the same extent in men, as supported by recent literature findings [27]. The decline in TMD prevalence with advancing age may be attributed to adaptive musculoskeletal changes, reduced exposure to contributing factors, or alterations in pain perception.

The differentiation of patients into subgroups based on the presence (Icd^i^) or absence (Icd^a^) of partial wisdom tooth inclusion provides further insight into TMD etiology. In younger patients (<25 years), the Icd^a^ subgroup accounts for a slightly higher proportion (53.8%) than Icd^i^ (46.2%), suggesting that third molar impaction is not the sole determinant of TMD onset. However, in the 26–30 age group, the proportion of Icd^i^ cases surpasses Icd^a^ (54.8% vs. 45.2%), indicating a potential peak period where impacted molars exacerbate TMD [27]. In the older age categories (31–35 and >36 years), the balanced distribution between Icd^i^ and Icd^a^ suggests that additional factors, such as degenerative changes or adaptive responses, contribute to TMJ pathology [27].

The mean Modified Helkimo Index values provide further evidence of the impact of third molar inclusion on TMD severity. Across all age groups, Icd^i^ scores remain consistently higher than Icd^a^, with the most pronounced differences observed in younger patients. In the <25-year group, the mean Helkimo index scores for Icd^i^ and Icd^a^ were 13.5 and 11.0, respectively (*p* = 0.045), indicating a statistically significant difference. A similar pattern was observed in the 26–30 age group (Icd^i^ = 10.0, Icd^a^ = 8.5, *p* = 0.038). However, the difference between Icd^i^ and Icd^a^ diminishes with age, with non-significant *p*-values in the 31–35 and >36-year categories (*p* = 0.061 and *p* = 0.072, respectively). This trend suggests that the influence of impacted molars on TMD symptoms may be transient, exerting more effect during early adulthood but becoming less relevant in older patients. Possible explanations [28] include spontaneous repositioning of impacted molars, structural adaptations of the TMJ, or resolution of acute inflammatory processes over time. The decline in TMD prevalence with advancing age may be attributed to adaptive musculoskeletal changes, reduced exposure to contributing factors, or alterations in pain perception [28]. While definitive causal relationships remain unestablished, evidence suggests that fluctuations in female sex hormones, particularly estrogen, may modulate pain perception in TMD patients. Studies have observed variations in TMD pain intensity corresponding with different menstrual cycles, indicating a potential link between hormonal changes and pain perception [27,28].

However, findings are not entirely consistent; some research has not found significant differences in TMD symptoms between women undergoing hormonal therapy and those not receiving such treatment. This variability suggests that while hormones may influence pain perception, they are likely one of multiple factors contributing to TMDs.

This study has clinical implications. The significant association between Icd^i^ and higher Helkimo Index scores in younger patients highlights the need for early intervention. Preventive extraction of partially included wisdom teeth may play a role in reducing the risk of TMD in susceptible individuals. The cross-sectional nature of the study does not allow for establishing a definitive causal relationship between impacted third molars and TMD, although our results suggest a strong association. Further longitudinal studies are necessary to confirm causality [27,29]. However, the diminishing impact on TMD severity in older patients emphasizes the necessity of a comprehensive assessment of all contributing factors beyond third molar inclusion.

Additionally, the marked female predominance in TMD prevalence underscores the importance of gender-sensitive treatment strategies. Due to the potential hormonal and behavioral influences on TMD pathogenesis, a multidisciplinary management approach is recommended. This could include pain-modulation strategies, behavioral therapy, hormonal considerations, traditional occlusal, and surgical interventions [26].

The study by Rani et al. [29] on dental students in Faridabad identified a 15% prevalence of TMDs, with a significant female predominance (79%), highlighting stress and hormonal influences. Key symptoms included joint sounds (7%), pain (3%), and muscle fatigue (2%). Clinical findings showed mouth-opening limitations in 6% and mandibular deviation in <1%. Both studies confirm a higher TMD prevalence in young women. However, our findings indicate a peak incidence under 25 years, potentially linked to wisdom tooth eruption, an aspect not addressed in the Faridabad study. This discrepancy suggests methodological or population-specific variations, warranting further research. The consistency of findings reinforces the Helkimo Index as a robust tool for TMD assessment in non-patient populations. The combined insights enhance epidemiological understanding and support the need for integrated clinical and diagnostic approaches.

The study by Al-Sanabani et al. [30] highlights the high prevalence of TMD in young individuals, with a progressive decline after 36 years. Women are more affected, likely due to hormonal influences, stress, and biological predisposition. The peak incidence occurs in the 18–25 age group, with symptoms often resolving over time due to physiological adaptations. This study and our research confirm a higher prevalence of TMD in young women and a decrease with age, but methodological differences exist. Unlike Al-Sanabani et al. [30], our study specifically examines the role of wisdom tooth eruption and provides a detailed analysis by gender and age, including female-to-male ratios and statistical significance. Further research integrating both approaches is needed to understand TMD etiology.

Our study’s data analysis (Table 4, Figure 3) provides a detailed overview of the distribution of patients with the clinical dysfunction index associated with impacted molars (Icd^i^) and those without impacted molars (Icd^a^) across different age groups. The results suggest a relatively balanced distribution between the two categories, with notable age-related differences in prevalence.

In patients under 25 years, Icd^a^ cases (53.8%) slightly outnumber Icd^i^ cases (46.2%), indicating that impacted molars are not the sole factor in TMDs at younger ages—other contributors include occlusal imbalances, muscular tension, trauma, or genetic predisposition [31]. However, the significant proportion of Icd^i^ suggests that partially impacted third molars may still increase TMD risk in this group.

Between 26–30 years, Icd^i^ cases become more prevalent (54.8%), suggesting an increasing contribution of impacted molars to TMDs, potentially due to symptom progression or complications like pericoronitis or occlusal changes [32]. In the 31–35 age group, the distribution is equal (50% Icd^i^, 50% Icd^a^), reflecting a multifactorial etiology where impacted molars are no longer a dominant risk factor. Physiological adaptations may also mitigate their effect on TMD [26].

In patients over 36 years, the total number of cases declines (8), with an equal Icd^i^–Icd^a^ distribution (50% each). This reduction may be due to physiological adaptation or prior treatments that reduced TMD prevalence [26]. However, the persistent presence of Icd^i^ cases suggests that impacted molars remain a contributing factor in older age groups; their role is less significant than other factors like joint wear or degenerative diseases.

The similar proportions of Icd^i^ and Icd^a^ across all age groups emphasize that while impacted molars play a significant role in TMDs, they are not the sole contributing factor. This observation is supported by the fact that, in each age category, at least half of the patients exhibit clinical dysfunctions unrelated to impacted molars. This highlights the importance of a holistic approach to diagnosis and treatment, considering factors like parafunctional habits, stress, and genetic predispositions [26,27,28,29].

The study by Alonso–Royo et al. [33] assesses the validity and reliability of the Helkimo Index for diagnosing TMDs in a cohort of 107 subjects (60 with TMD, 47 healthy). Results indicate high sensitivity (86.67%) and moderate specificity (68.09%), confirming its utility in distinguishing affected individuals. However, its lower complexity compared to the DC/TMD criteria considered the diagnostic gold standard [23] raises concerns about its specificity and precision. Inter-evaluator agreement was excellent for total scores but moderate for individual items, suggesting potential inconsistencies in component interpretation. Strong correlations with the Fonseca Anamnestic Index reinforce its validity, though its high sensitivity may increase false-positive rates. Our study parallels these findings, employing the Helkimo Index to analyze TMD prevalence and severity in patients with and without impacted molars. While Alonso–Royo et al. [33] do not stratify results by age, both studies emphasize multifactorial TMD etiology, including pain, mandibular restriction, and joint sounds. These insights underscore the Helkimo Index’s role as a practical screening tool while advocating for its refinement to enhance diagnostic accuracy.

The study by da Cunha et al. [34] evaluates the Helkimo Index and Craniomandibular Index (CMI) in diagnosing TMDs in rheumatoid arthritis (RA) patients. Among 80 subjects (70 RA, 10 controls), TMD prevalence was significantly higher in the RA group (98.6% vs. 80%), with restricted mandibular opening and joint sounds as predominant symptoms. Greater severity in RA patients highlights the impact of systemic disease on TMD progression, validating the Helkimo Index as a reliable assessment tool.

Our study and da Cunha et al. [34] employ the Helkimo Index in different contexts—impacted molars versus RA-related TMD. Despite distinct etiologies, findings converge on the role of systemic and local factors in TMD severity. Our study identifies a peak prevalence between ages 26 and 35, while da Cunha et al. [34] confirm symptom relevance in severity stratification.

Significant differences in Helkimo Index values (Icd^i^ and Icd^a^) (Table 5) are observed across age groups (<25, 26–30, 31–35, >36 years), reflecting the impact of impacted molars and age on TMDs.

The <25 group has the highest Icd^i^ (13.5), followed by 26–30 (10.0) and 31–35 (6.5), with the lowest in >36 (3.5), indicating more severe TMD in younger patients, likely due to occlusal changes and third molar impaction. Icd^a^ values are consistently lower (e.g., <25: 11.0 vs. 13.5 Icd^i^), reinforcing the role of impacted molars in symptom severity.

Variability is more in younger groups (e.g., <25: Icd^i^ max 22, Icd^a^ max 20; >36: Icd^i^ max 8, Icd^a^ max 10), suggesting a decline in symptoms with age due to structural adaptation or molar extraction. Statistical significance (*p* < 0.05) in <25 and 26–30 groups confirms a strong influence of impacted molars on TMD, which diminishes in older groups (*p* > 0.05).

Confidence intervals (95%) reveal more symptom variability in younger patients (<25: Icd^i^ 12.5–14.5, Icd^a^ 10.2–11.8), underscoring the need for individualized assessment. These findings confirm that impacted molars significantly exacerbate TMD in younger patients, while symptom reduction with age suggests that extraction helps prevention and management.

Renton et al. [35] highlight the role of impacted third molars in oral health and TMDs, linking their abnormal position to pericoronitis, chronic facial pain, and TMD. The study emphasizes how local inflammation increases pressure on the TMJ, leading to acute or chronic pain and restricted mandibular movement [33]. These findings align with our study, which shows higher Icd^i^ values in patients under 25, where impacted molars significantly worsen TMD symptoms. Renton et al. [35] detail pathological mechanisms such as chronic inflammation and muscle tension, correlating with our observed differences between Icd^i^ and Icd^a^ values. Both studies suggest that early extraction of impacted molars can reduce TMD severity and demonstrate that Icd^i^ values decrease with age, underscoring the need for early diagnosis and personalized intervention.

Hussain HA et al. [7] investigated the impact of third molar extraction on TMJ function using the Temporomandibular Joint Disability Index. Their study found a significant correlation between impacted molar extraction and functional difficulties such as chewing, speaking, and mouth opening, with common symptoms including pain, muscle fatigue, and restricted mandibular movement, particularly in younger patients. These findings align with our study, which reports higher Icd^i^ values in patients under 25, with the prevalence of severe clinical symptoms. Hussain et al. confirm that molar extraction can exacerbate TMD, especially in daily activities, reinforcing the observed differences between Icd^i^ and Icd^a^. Both studies highlight the role of impacted molars in increasing muscle tension and joint pain, influencing symptom variability. Additionally, ours shows a decrease in Icd^i^ values in older patients due to either molar extraction or structural adaptation. Hussain et al. [7] emphasize the need for proper extraction to prevent severe postoperative complications.

Marangos et al. investigated the association between third molar extraction and TMDs, identifying pain, restricted mandibular movement, and joint sounds. These can be exacerbated by surgical trauma, extraction difficulty, or patient predisposition to TMD. The study also highlights the influence of age, sex, and impaction severity on post-extraction complications [36].

Our study aligns with these findings, demonstrating a significant correlation between impacted molars and increased Icd^i^ values, particularly in younger age groups, reinforcing the role of third molars in TMD severity. Both studies observed a reduction of symptoms with age, suggesting that structural adaptation or molar extraction mitigates TMD severity. Marangos et al. further emphasize that optimal surgical interventions can minimize long-term complications [36].

This study presents several limitations that must be considered. The cross-sectional design precludes any inference of causality between impacted third molars and TMDs. Secondly, no prior sample-size calculation was performed, which might affect the generalizability and statistical power of the findings. The patient heterogeneity, including age differences, gender distribution, and clinical history, introduces potential biases that could influence results. The Helkimo Index, while appropriate for dysfunction severity evaluation, may lack the diagnostic precision of newer criteria like the DC/TMD. Future research should include longitudinal designs, larger and more homogeneous cohorts, and advanced diagnostic tools to validate these findings.

## 5. Conclusions

The Helkimo Index is useful for assessing TMD severity and correlating symptoms with impacted molars. Impacted third molars may contribute to TMD through chronic inflammation, occlusal changes, and muscle overload, potentially leading to pain, joint noises, and restricted mandibular movement. The incidence of included third molars peaks in patients under 25 and decreases after 36, suggesting biomechanical adaptation or tooth extraction. TMDs are more prevalent in women than in men. Significant differences between cases with or without third molar inclusion [support the association] of impacted molars with exacerbated symptoms, with peak severity observed in the 26–35 age group.

## Figures and Tables

**Table 1:** Helkimo Index Scale for Evaluating the Severity of TMDs [22].

Parameter	Description	Marks	Evaluation Scale (0–5 Points)
A. Limitation in the mandibular range of motion Dimension score	a. Maximum opening b. Maximum sliding to the right c. Maximum sliding to the left d. Maximum protrusion Summation of points	a. Upper incisal edge to lower incisal edge on the midline b. Maximum laterality (upper incisor line as reference) c. Same as item b d. Upper to lower incisal edge in maximum protrusive movement	a. 40 mm or more: 0 points (Normal opening) 30–39 mm: 1 point (Mild limitation) Less than 30 mm: 5 points (Severe limitation) b.7 mm or more: 0 points (Normal sliding) 4–6 mm: 1 point (Mild limitation) 0–3 mm: 5 points (Severe limitation) c,d. Same scoring as item b. - Normal mobility (0 points) = 0 - Mild impairment (1–4 points) = 1 - Severe impairment (5–20 points) = 5
B. Alterations in joint function	Opening and closing	Palpation and auscultation for joint noise, locking, or dislocation	- No deviation or sound = 0 points - Sounds or mandibular deviation = 1 point - Locking or dislocation (± sound) = 5 points
C. Pain on movement	Participant-reported pain		- No pain = 0 points - Pain in a single movement = 1 point - Pain in two or more movements = 5 points
D. Muscle pain	Palpation or functional manipulation	Check painful zones	- No pain in manipulation = 0 points - Pain in 3 zones = 1 point - Pain in 4+ zones = 5 points
E. TMJ pain	Palpation	Pain on periauricular or external auditory canal palpation	- No spontaneous or palpation pain = 0 points - Pain on unilateral/bilateral periauricular palpation = 1 point - Pain in both the external auditory canal and periauricular = 5 points

**Table 2:** The Modified Helkimo Index.

Score	Interpretation
0	No TMD
1–9	Mild TMD
10–19	Moderate TMD
20–25	Severe TMD

**Table 3:** Distribution by Age Categories and Gender.

F/M/Age Category	Female (*n*)	Male (*n*)	Female (%)	Male (%)	Female/Male Ratio	*p*-Value (*p* < 0.05)
<25 years	38	14	20.43	14.89	2.71	0.34
26–30 years	23	19	12.37	20.21	1.21	0.12
31–35 years	26	12	13.98	12.77	2.17	0.92
>36 years	6	2	3.23	2.13	3.00	0.89
Overall general	93	47	50.00	50.00	1.98	-

**Table 4:** Distribution of patients with Icd^i^/Icd^a^ according to age categories.

Icd^i^/Icd^a/^Age Category	Icd^i^ (n)	Icd^i^ (%)	Icd^a^ (n)	Icd^a^ (%)	Total (n)
<25 years	24	46.15	28	53.85	52
26–30 years	23	54.76	19	45.24	42
31–35 years	19	50.00	19	50.00	38
>36 years	4	50.0	4	50.00	8
Overall general	70	50.00	70	50.00	140

**Table 5:** Helkimo Index values (Icd^i^ and Icd^a^) across age groups with effect sizes.

Age Category	Mean Icdi	Min Icdi	Max Icdi	Mean Icda	Min Icda	Max Icda	*p*-Value	95% CI Icdi	95% CI Icda	Cohen’s *d*
<25 years	13.50	5.00	22.00	11.00	2.00	20.00	0.045	12.50–14.50	10.20–11.80	0.62 (medium)
26–30 years	10.00	4.00	17.00	8.50	1.00	18.00	0.038	9.10–10.90	7.80–9.20	0.55 (medium)
31–35 years	6.50	3.00	12.00	7.00	2.00	15.00	0.061	5.80–7.20	6.40–7.60	0.25 (small)
>36 years	3.50	1.00	8.00	4.50	0.00	10.00	0.072	2.90–4.10	3.90–5.10	0.37 (small)
**Total**	8.13	1.00	22.00	7.75	0.00	20.00	0.050	7.60–8.70	7.20–8.30	0.20 (small)

## Data Availability

Datasets are available from the authors upon reasonable request.
